# Colonoscopic or Capsule Fecal Microbiota Transplantation for Recurrent *Clostridioides difficile* Infection: A Prospective Cohort

**DOI:** 10.1016/j.gastha.2026.101051

**Published:** 2026-07-02

**Authors:** Dina Belhasan, Nataliia Kuchma, Monika Fischer, Jessica R. Allegretti, Colleen R. Kelly, Alexander Khoruts, Byron P. Vaughn

**Affiliations:** 1 Department of Medicine, University of Minnesota, Minneapolis, Minnesota; 2 Division of Gastroenterology, Hepatology, and Nutrition, University of Minnesota, Minneapolis, Minnesota; 3 Division Gastroenterology and Hepatology, Indiana University Health, Indianapolis, Indiana; 4 Division of Gastroenterology, Brigham and Women’s Hospital, Harvard Medical School, Boston, Massachusetts

**Keywords:** Microbiota-Based Therapeutics, Clinical Outcomes Research, Older Adults

## Abstract

**Background and Aims:**

Fecal microbiota transplant (FMT) is effective for preventing recurrent *Clostridioides difficile* infection (rCDI). Prior studies suggest that capsule and colonoscopic FMT administration are similarly effective, although the studies may be limited by small sample size or selection bias. The aim of this study was to compare the CDI recurrent rates following capsule or colonoscopic FMT.

**Methods:**

Prospective cohort of patients with rCDI treated with capsule or colonoscopic FMT between 7/1/19 and 11/29/23. This study’s primary outcome was 1-month CDI recurrence rates. Predictors of recurrence were assessed using multivariate logistic regression.

**Results:**

Overall, 652 individuals received FMT (421 capsule, 223 colonoscopy). Those receiving capsule FMT were older (median 70 vs 59 years, *P* < .001). At 1 month, CDI recurrence was higher with capsule compared to colonoscopic FMT (18% vs 7.4%, *P* = .001). On multivariate analysis, capsule FMT was associated with increased rCDI risk (adjusted odds ratio [OR]: 2.37, 95% confidence interval [CI]: 1.16–5.24, *P* = .023). Stratified analyses by age showed capsule FMT increased recurrence risk only in patients ≥65 years (OR: 2.7, 95% CI: 1.2–6.4), but not in those <65 years (OR: 2.7, 95% CI: 1.2–6.4). Beyond 1-month, non-CDI antibiotic exposure became the predominant predictor of recurrence.

**Conclusion:**

FMT by capsule may be associated with higher CDI recurrence compared to FMT by colonoscopy. This effect appears be greater in individuals older than 65 years, thus highlighting the importance of individualized treatment decisions that balance FMT efficacy with procedural risks. Development of microbiota-based therapeutics for CDI should account for the impact of administration route on efficacy.

## Introduction

*Clostridioides difficile* is a gram positive, spore-forming bacteria that causes infectious colitis in humans. *C difficile* infection (CDI) typically arises when the commensal intestinal microbiota is disrupted (dysbiosis), most often following antibiotic exposure. Despite appropriate antibiotic treatment, CDI recurrence rates remain high, with the risk increasing after each subsequent episode.^1^^,^^2^ Several risk factors have been identified, with non-CDI antibiotic exposure being the most significant.^3^ Other well-established risk factors include advanced age, inflammatory bowel disease, immunosuppression, chronic kidney disease, and solid organ transplantation.^1^^,^^4^, ^5^, ^6^

Fecal microbiota transplantation (FMT) has an estimated success rate of ∼80% for preventing additional CDI recurrences.^7^^,^^8^ The route of FMT administration influences efficacy: colonoscopy and oral capsules demonstrate the highest success rates, while nasoenteric and enema delivery show reduced efficacy.^9^^,^^10^ Colonoscopy and capsule administration appear similarly effective with a small randomized controlled trial reporting no difference in 12-week CDI recurrence between the 2 methods.^11^

In an earlier version of our national CDI cohort, no significant difference was observed between capsule and colonoscopy administration.^8^ However, the point estimate for capsule efficacy was lower at 1 month (84% vs 91%, *P* = .12).^8^ A potential explanation for this difference was selection bias, as older patients were more likely to receive capsule FMT. Additionally, the earlier cohort may have been underpowered to detect a difference between FMT route.

The present study aims to compare CDI recurrence rates following colonoscopic vs capsule FMT in a larger national cohort and evaluate the influence of traditional risk factors, including age and sex, on route-specific effectiveness.

## Methods

We analyzed a prospective cohort of patients with recurrent CDI (rCDI) who were treated with FMT between July 1, 2019, and November 29, 2023, across 4 academic centers (University of Minnesota, Indiana University, and Harvard-affiliated centers) and 2 community practice groups (MNGI Digestive Health and Ventura). Two FMT formulations were available to participating centers: frozen liquid for colonoscopic administration and freeze-dried encapsulated preparation. The number of bacteria (∼5 × 10^11^) was the same in the 2 preparations. Further details regarding FMT preparation and study methods are available as published in our prior cohort; no changes were made to the original protocol.^8^

FMT preparations were provided free of charge, and the choice of administration route was left to the discretion of the treating physicians, with the stipulation that baseline and outcome data be provided. Antibiotic treatment was recommended to be discontinued 1 to 3 days prior to bowel preparation in the colonoscopic FMT group and 2 to 3 days prior to administration in the capsule FMT group. No specific recommendations were made on bowel purgative prior to capsule FMT administration. Patient consent was obtained by treating physicians in accordance with Food and Drug Administration (FDA) guidance. The study was approved by the University of Minnesota institutional review board.

### Data Collection

Deidentified data regarding baseline patient characteristics and outcomes were collected from treating providers using the Research Electronic Data Capture. In addition to baseline patient characteristics, follow-up data were collected at 1 week and at 1, 2, 6, and 12 months through automated surveys sent to treating physicians. The 1-week survey was limited to CDI recurrence and adverse events assessment. Non-CDI antibiotic use was assessed starting at 1 month and at each subsequent survey. Analyses were restricted to individuals with available data at each time point; missing outcomes were not imputed or assumed to represent treatment success. When survey data were unavailable, chart review was performed when feasible.

### Outcomes

The outcome of interest for this study was CDI recurrence at 1 month. One month was chosen as there was less non-CDI antibiotic administration over this time (which is a known risk for CDI recurrence post-FMT) and to minimize missing data. Diagnosis of CDI recurrence post FMT was defined as cases meeting all 3 criteria: 1) treating provider interpretation of CDI recurrence, 2) positive supportive stool testing per local laboratory process, and 3) resumption antibiotic therapy directed against *C difficile*. CDI recurrence was assessed at 1 week, 1 month, 2 months, 6 months, and 12 months.

### Sample Size and Power

In our prior cohort, we observed a 7% absolute treatment difference in CDI recurrence at 1 month between capsule and colonoscopic administered FMT (84% v 91%, respectively). At that time, 170 individuals received capsule FMT and 96 individuals received colonoscopic FMT. The post hoc power to detect a 7% difference was 39%. Assuming a 7% treatment difference, and a similar proportion of individuals receiving capsule and colonoscopic FMT, we estimated needing 446 individuals receiving capsule FMT and 252 individuals receiving colonoscopic FMT to have 80% power to detect a 7% difference in the CDI recurrence rate at 1 month.

### Statistical Analysis

Predictors of CDI recurrence following FMT were assessed using recurrence data from within 1 month. Patients with missing data were excluded from the analysis. Only first CDI recurrences were counted as recurrences. *P* values were calculated using Fisher's exact test with a *P* < .05 considered as significant. Multivariate logistic regression was used to identify independent predictors of CDI recurrence. Based on earlier analysis of our cohort, we observed that older individuals were preferentially administered capsule FMT. As age is a known risk factor for CDI recurrence, we explored the effect of significant risk factors on multivariate regression above and below age 65. Effect modification was assessed for on relevant first order interactions. Odds ratios (ORs) for recurrence were calculated for each variable using bivariate logistic regression. Results are presented with 95% confidence intervals (CIs). All analyses were conducted in RStudio (version 4.4.3).

## Results

### Baseline Patient Characteristics

A total of 652 individuals received FMT: 421 via capsule and 223 via colonoscopy. Eight individuals received nonstandard FMT administration and were excluded from analysis: these included orally administered liquid suspensions, G-tube administrated products, and combination capsule and colonoscopic FMT, leaving 644 individuals in the final analysis set. Consistent with our previous cohort, more individuals received capsule FMT than colonoscopic FMT (65% vs 35% respectively). Baseline characteristics of patients in the colonoscopic and capsule FMT groups are presented in Table 1. Individuals receiving capsule FMT were older than those receiving colonoscopic FMT (median age 70 vs 59, *P* < .001) and less likely to be immunocompromised (16% vs 27%, *P* < .001). There were numerically more individuals with inflammatory bowel disease (IBD) in the colonoscopic FMT group, although this was not statistically significant. Otherwise, the groups appeared similar in CDI prior treatment and baseline characteristics.

**Table 1:** Patient Demographics at the Time of Receiving Fecal Microbiota Transplantation

Characteristic	Total (N = 644)	Capsule (N = 421)	Colonoscopy (N = 223)	*P* value
Female sex	472 (73%)	312 (74%)	160 (72%)	.5
Age ≥65 y	363 (56%)	267 (64%)	96 (43%)	<.001
White race	603 (94%)	393 (93%)	210 (95%)	.3
Immunocompromised	117 (18%)	60 (16%)	57 (27%)	<.001
Proton pump inhibitor use	159 (25%)	105 (25%)	54 (24%)	.9
Inflammatory bowel disease	63 (10%)	38 (8.8%)	27 (13%)	.2
Prior CDI hospitalization	215 (35%)	136 (34%)	79 (37%)	.5
CDI episodes in prior year				.3
No recurrences	37 (6.0%)	23 (5.7%)	14 (6.3%)	
1	53 (8.8%)	37 (9.2%)	16 (7.3%)	
2	205 (33%)	125 (31%)	80 (37%)	
3	159 (26%)	102 (25%)	57 (27%)	
>3	190 (31%)	134 (32%)	56 (25%)	
Prior CDI therapy				
Metronidazole	140 (22%)	94 (22%)	46 (21%)	.7
Vancomycin (10–14 d)	553 (86%)	359 (85%)	194 (87%)	.6
Vancomycin (>4 wk)	414 (64%)	274 (65%)	140 (63%)	.6
Fidaxomicin	248 (39%)	168 (40%)	80 (36%)	.5
Rifaximin	93 (14%)	65 (15%)	28 (13%)	.9
Bezlotoxumab	29 (4.5%)	22 (5.2%)	7 (3.1%)	.3
Prior FMT	108 (18%)	71 (18%)	37 (17%)	>.9
Comorbid condition				
Dialysis	17 (2.6%)	8 (1.9%)	9 (4.0%)	.12
Decompensated liver disease	11 (1.7%)	6 (1.4%)	5 (2.2%)	.5
Cognitive impairment	37 (5.7%)	22 (5.2%)	15 (6.7%)	.5
Neuromuscular impairment^a^	29 (4.5%)	21 (5.0%)	8 (3.6%)	.5

Note. Data are presented as number (%).

a Neuromuscular impairment is defined as any condition that could impair normal mobility and getting the bathroom.

### *Clostridioides difficile* Infection Recurrence Rates by Fecal Microbiota Transplantation Route

Five hundred fourteen (80%) individuals had data at the week 1 assessment. The week 1 CDI recurrence rate was significantly higher among those who received capsule FMT compared to colonoscopic FMT (9.2% v 3.2%, *P* = .01). At 1 month, data were available for 505 individuals (78.4%). During this period, an additional 28 individuals who received capsule FMT developed CDI recurrence while only an additional 7 recurrences were noted in the colonoscopic group, resulting in 1-month CDI recurrence 18% for those who received capsule FMT and 7.4% for those who received colonoscopic FMT (*P* = .002) (Table 2).

**Table 2:** Cumulative *Clostridioides difficile* Recurrence Rate at Each Follow-Up

CDI recurrence	Overall (N = 644)	Capsule (N = 421)	Colonoscopy (N = 223)	*P* value
1 mo	71/505 (14%)	58/329 (18%)	13/176 (7.4%)	.002
2 mo	90/503 (18%)	66/330 (20%)	24/173 (14%)	.09
6 mo	106/403 (26%)	78/267 (29%)	28/136 (21%)	.06
12 mo	116/318 (36%)	84/209 (40%)	32/109 (29%)	.06

Note. Data are presented as number (%).

The cumulative CDI recurrences rates at 2, 6, and 12 months were not statistically significant between capsule and colonoscopy administered FMT, although numerically favored colonoscopy FMT administration (Table 2).

### Predictors of *Clostridioides difficile* Infection Recurrence by 1 Month

In addition to FMT route, independent patient predictors of CDI recurrence at 1 month (Table 3) included age ≥65 years (*P* = .007), history of prior CDI-related hospitalizations (*P* = .02), ≥2 CDI recurrences in the past year (*P* = .048), and dialysis (*P* = .005). Proton pump inhibitor (PPI) use trended higher in those with CDI recurrence but did not reach significance (36% vs 24%, *P* = .06).

**Table 3:** Patient Predictors of *Clostridioides difficile* Recurrence 1 Month Postfecal Microbiota Transplantation

Characteristic	CDI recurrence (N = 73)	No CDI recurrence (N = 439)	*P* value
Female sex	52 (72%)	319 (73%)	.9
Age ≥65 y	53 (73%)	242 (55%)	.007
White race	71 (97%)	412 (94%)	.4
Immunocompromised	12 (17%)	79 (18%)	.9
Current PPI use	26 (36%)	107 (24%)	.06
Inflammatory bowel disease	6 (8.6%)	49 (11%)	.7
Prior CDI hospitalization	33 (49%)	141 (33%)	.02
≥2 CDI recurrences in past year	65 (93%)	362 (84%)	.048
Prior FMT	14 (20%)	79 (18%)	.7
Dialysis	6 (8.2%)	7 (1.6%)	.005
Decompensated liver disease	0 (0%)	9 (2.1%)	.4
Cognitive impairment	4 (5.5%)	32 (7.3%)	.8
Neuromuscular impairment	10 (14%)	48 (11%)	.5
Non-CDI antibiotics	5 (8.8%)	18 (4.1%)	.2

Note. Data are presented as number (%).

The rate of non-CDI antibiotics administered within 1 month was similar in both groups (4.5% for capsule FMT and 3.4% for colonoscopic FMT, *P* = .5). Administration of non-CDI antibiotics within 1 month did not predict CDI recurrence overall (*P* = .09). However, excluding individuals who experienced CDI recurrence by 1 week (when additional antibiotic use was not assessed), non-CDI antibiotics were associated with CDI recurrence at 1 month (*P* = .01).

On multivariate analysis, age >65, dialysis use, and FMT administration (capsule vs colonoscopy) remained significantly associated with CDI recurrence at 1 month (Table 4). The adjusted OR for capsule FMT on 1 month CDI recurrence was 2.4 (95% CI: 1.2–5.2, *P* = .02).

**Table 4:** Multivariate Analysis of *Clostridioides difficile* Infection Recurrence at 1 Month

Characteristic	Univariate OR (95% CI)	*P* value	Multivariate OR (95% CI)	*P* value
Female	0.99 (0.57, 1.78)	>.9	1.05 (0.54, 2.15)	.9
Age ≥65 y	2.18 (1.27, 3.90)	.006	1.66 (0.87, 3.30)	.1
Dialysis	5.63 (1.76, 17.5)	.003	9.44 (2.75, 32.3)	<.001
Prior CDI hospitalization	1.78 (1.05, 3.01)	.03	1.24 (0.65, 2.31)	.5
≥2 CDI recurrences in past year	2.43 (1.03, 7.13)	.07	1.40 (0.57, 4.21)	.5
Non-CDI antibiotics	2.42 (0.77, 6.43)	.1	2.71 (0.82, 7.77)	.08
FMT administration route	2.68 (1.47, 5.25)	.002	2.37 (1.16, 5.24)	.02

As older patients were more likely to receive capsule FMT, we explored the impact of capsule FMT administration on those above and below age 65. Among patients ≥65 years, capsule FMT was associated with a 2.7-fold increased CDI recurrence risk compared to colonoscopy (95% CI: 1.2–6.4, *P* = .02), whereas no significant association was observed in patients <65 years (OR: 1.9, 95% CI: 0.7–5.3, *P* = .2). However, although stratified analyses suggested a stronger association in older patients, the interaction term was not statistically significant in the regression model, and thus formal evidence of effect modification was not established. Dialysis use remained a significant predictor of CDI recurrence in individuals over and under age 65.

### Predictors of *Clostridioides difficile* Infection Recurrence at Later Months

Baseline patient characteristics and FMT route were not associated with CDI recurrence beyond 1 month, apart from age at 1 year (*P* = .04). However, after adjusting for non-CDI antibiotic use, age was no longer significant. The major risk factor for CDI recurrence after 1 month was additional non-CDI antibiotic use (at 2 months 24% vs 11%, *P* = .011; at 6 months 50% vs 25%, *P* = .003, at 12 months 89% vs 38%, *P* < .001) (Table 5).

**Table 5:** *Clostridioides difficile* Recurrence by Additional Antibiotic Administration

Non-CDI antibiotic administration within each time point	CDI recurrence (N, %)	No CDI recurrence (N, %)	*P* value
Within 1 mo	5 (8.8%)	18 (4.1%)	.2
Within 2 mo	12 (24%)	38 (11%)	.01
Within 6 mo	19 (50%)	64 (25%)	.003
Within 12 mo	31 (89%)	64 (38%)	<.001

## Discussion

We found that FMT administration orally via capsule had a 10% increased risk of CDI recurrence at 1 month compared to colonoscopic FMT administration. This effect was persistent after adjusting for potential confounders. Stratification by age revealed that this association was limited to patients aged 65 years and older, a group both more likely to receive capsule FMT and more prone to CDI recurrence. These findings suggest that capsule FMT may be less effective in older adults for preventing CDI.

This study identified differences in efficacy that were not observed in our prior cohort, which included approximately half as many patients. Both the current and previous cohorts had a comparable proportion of patients receiving capsule and colonoscopic FMT at an approximate 2:1 ratio, and the study protocol remained unchanged. Baseline differences between groups were generally consistent across cohorts, apart from IBD prevalence, which was higher in the colonoscopic group in our previous study but similar between groups in the present cohort. Overall, the prior analysis was likely underpowered to detect a difference of 7%. Our current analysis noted a larger difference in CDI recurrence rate between capsule and colonoscopic FMT (10.6%). Accounting for this, our post hoc power based on our sample size was >80%.

A prior randomized controlled trial comparing capsule and colonoscopic FMT found capsule administration to be noninferior.^11^ This study included approximately 120 participants randomized to either capsule or FMT colonoscopy with only 2 recurrences in each arm. Based on our assumptions and experience, this study was likely underpowered to detect a treatment difference between capsule and colonoscopic FMT. Additionally, the median age of participants in the randomized controlled trial was <60 years. Thus, the study may have not included sufficient older individuals where a difference in capsule or colonoscopy FMT is significant. The dose of capsule FMT used in this study was 40 capsules prepared from 100 g of stool, which is likely higher than the standard FMT capsule dose in our cohort. We previously investigated dosing of our FMT capsule formulation and did not find a benefit with higher numbers of capsules.^12^ Nonetheless we cannot rule out the need for a higher or repeated capsule dose, particularly in older individuals.

Significant differences in FMT CDI recurrence rates between capsule and colonoscopy administration were only observed at 1 month. One possible explanation relates to microbiota engraftment kinetics. Colonoscopic FMT typically achieves donor microbiota engraftment within 48 h, whereas capsule FMT engraftment is delayed 2 to 4 weeks.^13^, ^14^, ^15^, ^16^, ^17^ Delayed engraftment may therefore account for our findings. No significant differences in CDI recurrences were noted between colonoscopy or capsule FMT after 1 month, although recurrence rates remained numerically higher in the capsule group. Most recurrences occurred within 1 month; therefore, we may still be underpowered to detect differences at later time points. Moreover, after 1-month, non-CDI antibiotics became the predominant risk factor for recurrence consistent with prior literature.^3^^,^^18^ It is likely this reduces the relative impact of FMT route.

Consistent with prior literature, we identified age as a key predictor for CDI recurrence post FMT.^19^^,^^20^ Older patients may be more vulnerable to delayed engraftment due to increased microbiome fragility and a diminished ability to sustain the microbial diversity typically seen in younger individuals. These age-related microbiome changes are associated with functional impairments such as reduced short-chain fatty acid metabolism, decreased saccharolytic and proteolytic fermentation, increased intestinal permeability, and elevated inflammatory markers.^21^, ^22^, ^23^, ^24^, ^25^, ^26^, ^27^, ^28^ However, what is novel in our cohort, is that the impact of age was different for those receiving capsule or colonoscopic FMT. PPI use was more common among those with recurrence (36% vs 24%) and approached statistical significance (*P* = .06), demonstrating a trend consistent with prior literature. A large meta-analysis found an absolute risk increase of 6% for rCDI with PPI use.^29^ Our cohort was likely underpowered to detect a significant PPI risk. Overall, our data support that unnecessary PPI use should be avoided in people with CDI.

Among patients aged ≥65 years, capsule FMT was associated with a significantly higher risk of recurrence, whereas no difference was observed in patients <65 years. There are a few possible explanations for our finding. Differences in engraftment kinetics between capsule and colonoscopic FMT may make elderly individuals more challenging targets for capsule-based engraftment. For example, in addition to having a more protracted engraftment course (as previously discussed), capsule FMT leads to lower abundances of donor bacteria after 1 month relative for colonoscopic FMT even in FMT responders.^14^ Accordingly, the interplay between the poor temporal stability of the aging microbiome and the protracted engraftment course that occurs with capsule FMT together may result in the lower successful engraftment with capsule FMT specifically.^21^^,^^22^

Another plausible explanation concerns logistical differences between administration of capsule and colonoscopic FMT; specifically, most capsule recipients did not undergo bowel purgative. Bowel purgative is thought to increase the opportunity for donor engraftment by purging of the host both bacteria and residual vancomycin.^14^ However, the lack of bowel purgative did not impact capsule FMT efficacy in younger patients compared to colonoscopy FMT. However, it is conceivable that lack of bowel purgative created more significant engraftment difficulty in the elderly group undergoing capsule FMT. In our cohort, the dose was standardized for both the capsule and colonoscopic preparation of FMT material. It may be that oral dosing requires a higher dose, or additional dosing to improve efficacy. Ongoing questions therefore remain about the optimal strategy (bowel purgative or not), dose, and frequency of oral microbiota products.

Despite an apparent difference in recurrence risk across age strata, formal testing of interaction did not demonstrate significant effect modification. Consequently, the stratified findings should be interpreted with caution and do not confirm age as a moderator of treatment response.

It is not clear if our findings directly apply to FDA approved microbiota therapeutics. Existing FDA approved therapies are donor derived similar to FMT. The oral microbiota therapeutics, fecal microbiota spores, live-brpk (Vowst), is different from FMT in that it consists of purified spores. Unlike our FMT capsule administration, Vowst does include a bowel purgative prior to ingestion and 3 consecutive days of dosing. The estimated efficacy of Vowst in individuals aged ≥65 years is 83% at 2 weeks.^30^ This is similar to our single-dose capsule FMT effectiveness rate of 82%. There is no comparable colonoscopy administration for Vowst. Fecal microbiota, live-jslm (Rebyota) is a minimally manipulated, donor derived live biotherapeutic administered via retention enema. Enema administration of FMT appears to be less consistent than colonoscopy independent of age.^9^ In individuals aged ≥65 years who received Rebyota, the treatment success rate was 70% at 8 weeks. It is unknown if this lower point efficacy is related to microbial dose, administration route, or the study population.

Dialysis was also found to be a significant independent predictor of CDI recurrence in our cohort. Both chronic kidney disease and end-stage renal disease are well-established risk factors for rCDI, with further increased risk in those with concurrent dialysis dependence.^6^^,^^31^ Our finding that dialysis is a significant risk factor for rCDI is consistent with previous literature and underscores the extent to which hemodialysis increases CDI recurrence risk. The mechanism remains unclear; however, in our data, this could not be explained solely by additional non-CDI antibiotic exposure. Other drivers of intestinal dysbiosis may be present in this patient population.

This study has several limitations. As an observational cohort, selection bias and residual confounding remain possible explanations for our findings. Data collection was tied to clinical assessments, and when these were unavailable, providers abstracted information from chart review. Moreover, CDI diagnoses were based on local laboratory practices, which may vary, and we did not capture details of specific laboratory tests used. Despite these limitations, our study also has notable strengths. While the observational design limits direct comparison of treatment modalities in a standardized way, it reflects real-world clinical practice more closely. Additionally, the large sample size enhances statistical power, and recruitment across diverse academic and community sites spanning multiple geographic regions strengthens generalizability.

## Conclusion

In summary, we found that that capsule FMT has a higher CDI recurrence rate compared to colonoscopic FMT, particularly in older individuals. This finding is clinically important, as capsule FMT provides a less invasive alternative and avoids the procedural risks of colonoscopy, which may be higher in older patients. These results underscore the importance of careful clinical decision-making, weighing the risks associated with colonoscopy against the potentially reduced efficacy of capsule FMT when treating older patients with rCDI. These findings are also important to the development of future microbiota therapeutics, which should incorporate bowel purgative and administration route into their design to optimize efficacy across different patient populations.
